# Integrated imaging in rare eyelid lesions: a case report of palpebral dirofilariosis

**DOI:** 10.1186/s12348-025-00536-z

**Published:** 2025-10-21

**Authors:** Luca Tinunin, Filippo Ugolini, Dario Giattini, Nicola Santoro

**Affiliations:** 1 Section of Anatomic Pathology, Department of Health Sciences, University of Florence, Florence, Italy; 2 Section of Anatomic Pathology, Careggi University Hospital, Florence, Italy; 3 Unit of Ocular Oncology, Department of Surgery and Translational Medicine, Careggi University Hospital, Florence, Italy; 4 Ophthalmic Surgery Unit, “S. Giuseppe Hospital”, Empoli, FI Italy

**Keywords:** Integrated imaging, Ophthalmic pathology, Dirofilariosis, Palpebral parasitosis

## Abstract

**Introduction:**

Integrated evaluation of clinical records, radiologic imaging, and histologic slides was key in the diagnosis of a rare ophthalmic infection such as *Dirofilaria repens*, an emerging zoonosis in many Mediterranean regions, where it is endemic in cats and dogs and can spread to humans through mosquitos of the genera *Anopheles*,* Aedex* and *Culex.*

**Case report:**

We present the case of a 64-year-old male with a subcutaneous nodule at the right medial canthus. Surgical excision was performed under the clinical suspicion of a sebaceous cyst, and the specimen was submitted for pathological examination. Histological analysis revealed a partially necrotic abscess with numerous eosinophils and multiple cross-sections of the body of a parasite. The integrated evaluation of CT, MRI and histologic sections were consistent with the diagnosis of infestation by a female *Dirofilaria repens*.

**Discussion:**

In this report we discussed an illustrative case of *Dirofilaria* infestation, an uncommon condition, and illustrate the benefits of an integrated approach to the care of ophthalmic lesions. Indeed, direct access to digitalized images of different modalities is beneficial in reducing delays, increasing accuracy, and is beneficial for prompt medical care and accurate diagnosis. The rising incidence and the possibility of serious complications, especially for the less frequent localizations more typical of species other than *Dirofilaria repens*, translates the need for a more widespread knowledge of these zoonoses.

## Introduction

As imaging tools advance in resolution and accessibility, the opportunity for image integration becomes increasingly available and useful. This approach is particularly valuable in high complexity disciplines such as ophthalmology that employ multiple imaging modalities [10], allowing for all involved in the care of patients to always access relevant imaging and clinical records, thus reducing delays and preventing mistakes [4].

Furthermore, integrated systems enable more convenient consultations by facilitating real-time collaboration between specialists, regardless of their physical location, thus breaking down barriers to expert insights and improving diagnostic accuracy [15].

This report aims to illustrate an illustrative case of a rare disease in ophthalmic pathology, in which the collaboration between surgeon and pathologist was key to reaching the correct diagnosis.

Ophthalmic parasitosis caused by helminths in the genus *Dirofilaria* are exceedingly rare in humans, yet, growing evidence suggests that these infections are on the rise in southern and eastern Europe, as well as in southern Asia, and the USA [18, 19]. Worldwide there have been 1782 cases reported [16]. The mediterranean basin is the area with the highest incidence in Europe, with Italy (336 cases) and France (91 cases) at the forefront [16].

*Dirofilaria repens* infections often present as subcutaneous eyelid nodules [2, 5], but a few recent reports have started documenting microfilaremia in humans [20]. Histological examination and description of the parasite’s distinctive morphological features is usually sufficient for a diagnosis [13]. If blood tests are conducted, specific serum IgGs can be documented by ELISA, PCR can confirm the identification of the parasite as *D.repens*, and microfilaremia can be documented using a modified Knott’s test [20, 22].

## Case report

A 64-year-old male veterinary surgeon was referred to our Oculoplastic Service (Careggi University Hospital, Florence, Italy) in September 2023, for the evaluation of a painless nodule at the medial canthus of the right eye (RE). The lesion appeared 4 months prior and was initially treated as dacryocystitis with oral antibiotics, with no benefit.

He did not report any recent travels to Africa or Asia, but he was on a trip to Rimini (Emilia Romagna, Italy) the month before the onset of the lesion, during which he experienced a tingling sensation at the upper eyelid of the RE, as well as itching and redness.

**Fig. 1:**
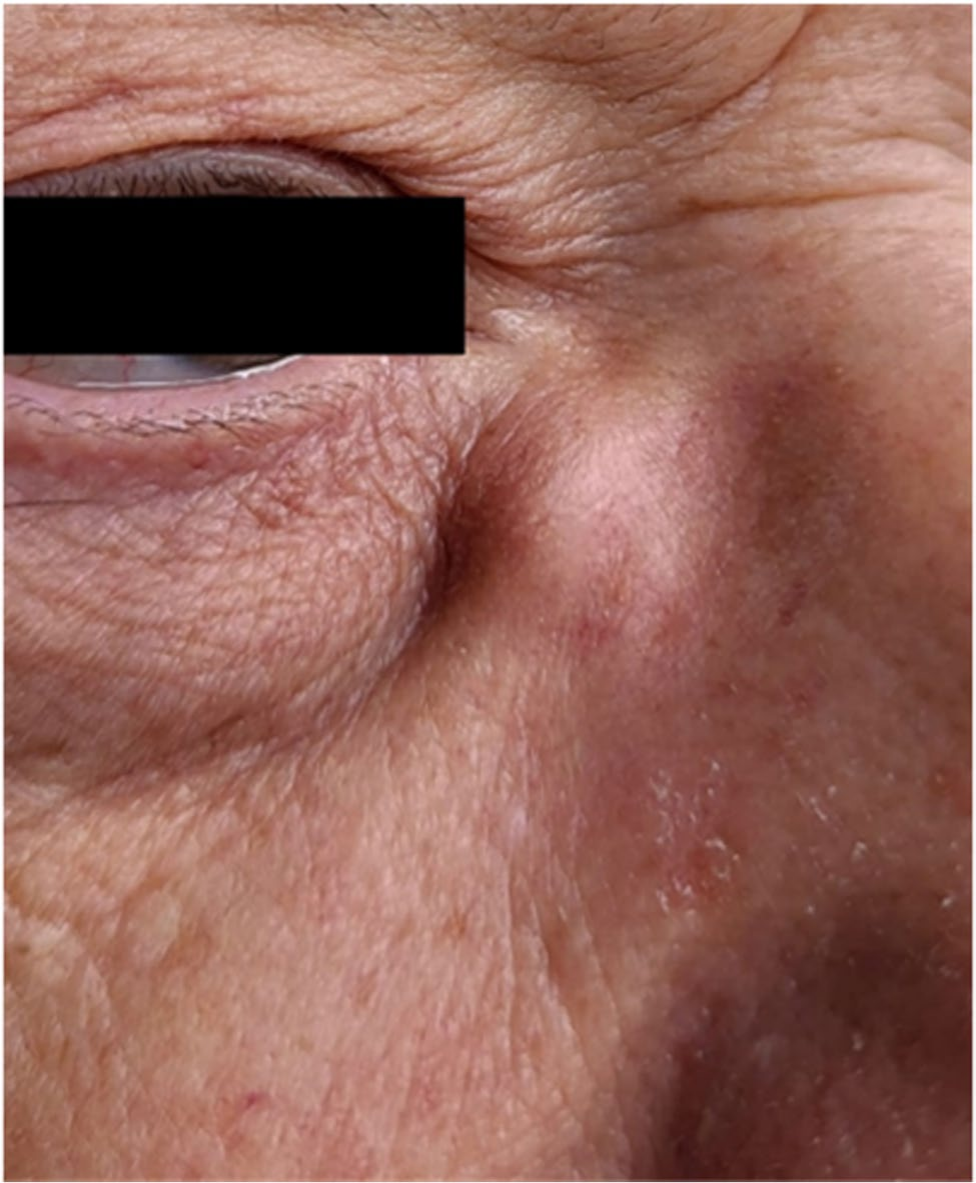
A subcutaneous non-mobile nodule at the right medial canthus. The clinical findings supported the initial diagnosis of dacryocystits

A complete ophthalmological and adnexal examination revealed a swelling lesion at the medial canthus of the RE, without signs of inflammation. The nodule was adherent to deep planes and the overlying skin was not erythematous or ulcerated (Fig. 1).

**Fig. 2:**
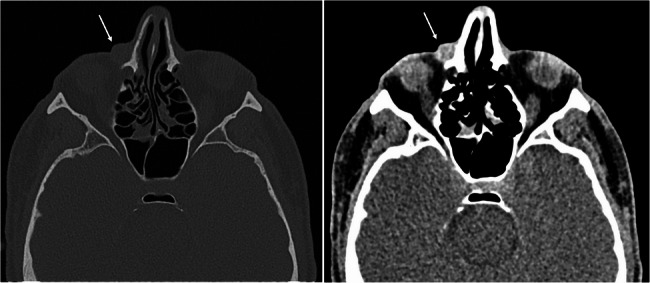
Facial computed tomography (CT) scan, showing a slightly hyperintense preseptal nodule (arrow) overlying the right medial canthal tendon, and slight air entrapment in the controlateral (left) lacrimal sac

No other alterations of the eye or the lacrimal apparatus were documented. A facial computed tomography (CT) scan showed a slightly hyperintense preseptal nodule (Fig. 2).

**Fig. 3:**
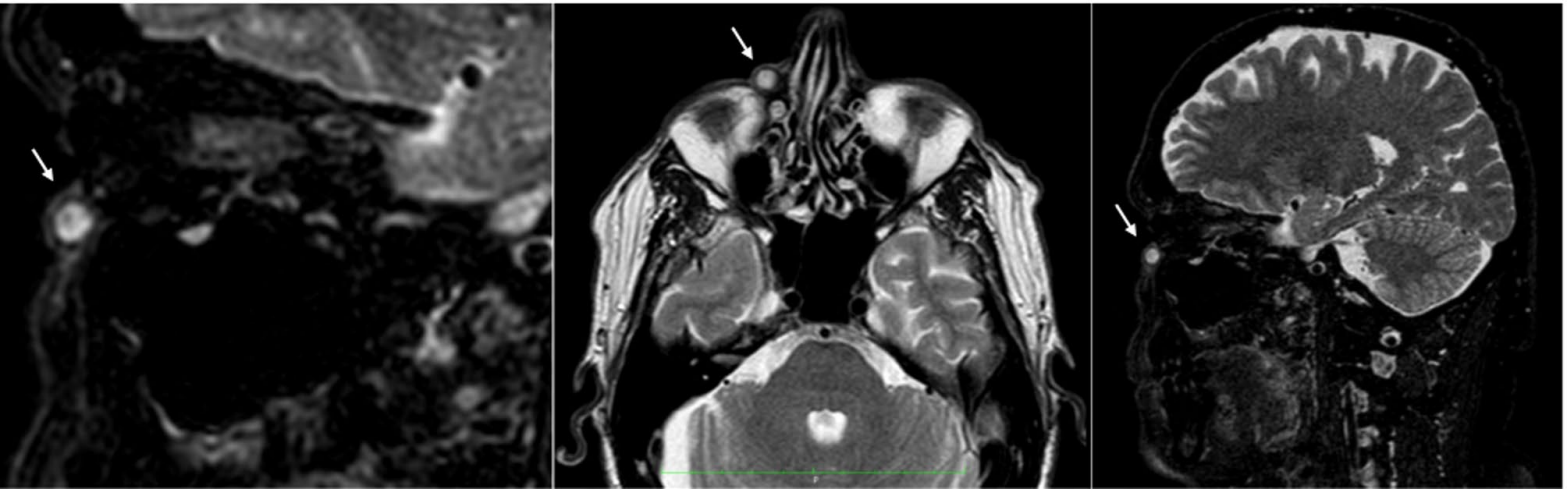
MRI (T2-weighted axial image) round lesion measuring 8 × 8 × 9 millimeters, located in the right inferomedial periorbital area (white arrow), adjacent to the frontal process of the maxillary bone. The lesion was characterized by well-defined and encapsulated margins (thickness of about 2 millimeters), hyperintense signal in T2 with marked enhancement after contrast administration, and a slight faded alteration of the perilesional soft tissue signal, consistent with perilesional edema

Subsequently, Magnetic resonance imaging (MRI) was also obtained to further characterize the lesion (Fig. 3).

These clinical and imaging findings of an inflammatory-infectious lesion, located in the right periorbital area, overlying the medial canthal tendon, were deemed insufficient for a conclusive diagnosis and could not completely exclude from the differential diagnosis a neoplastic lesion. Therefore, we elected to perform an excisional biopsy.

The excised nodule, measuring 1.2 cm at its greatest dimension, was sent to the department of pathology for examination. The surgical fragment measured 1.1 × 0.8 × 0.5 cm; the nodule was then divided in half but showed no salient macroscopic features. Stained tissue sections were digitally scanned at ×400 magnification with a microscope slide scanner, Aperio AT2 platform (Leica Biosystems, Germany), into whole slide digital images (WSI).

**Fig. 4:**
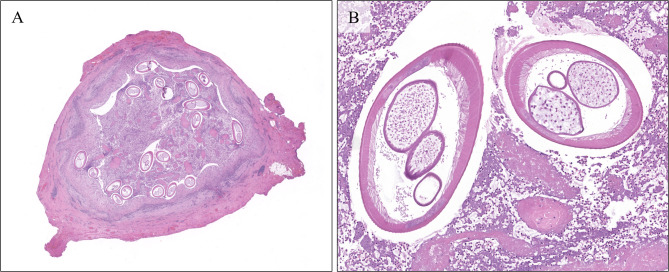
**A** Low magnification view of the subcutaneous nodule, showing an abscess surrounded by fibrotic reaction with plasma cells and histiocytes, and containing multiple cross-sections of the parasite. H&E. **B** 10X magnification showing the contents of the abscess. Two cross-sections, one transversal and the other at an angle, of the body of the worm. The parasite is surrounded by necrotic debris, with fibrin deposits and numerous inflammatory cells, chiefly eosinophil, and neutrophil granulocytes. H&E

Histopathological examination revealed an abscess with abundant necrotic debris, globular fibrin masses and numerous inflammatory cells, namely eosinophil and neutrophil granulocytes, with accompanying histiocytes, plasma cells and lymphocytes, surrounding multiple cross sections of the body of a parasite (Fig. 4).

The morphological features, as illustrated in the previous sections, were highly specific, representing one of the most common microscopic findings of this condition *(*Pampiglione et al., [12] and the diagnosis of subcutaneous infection by *Dirofilaria repens* was made.

**Fig. 5:**
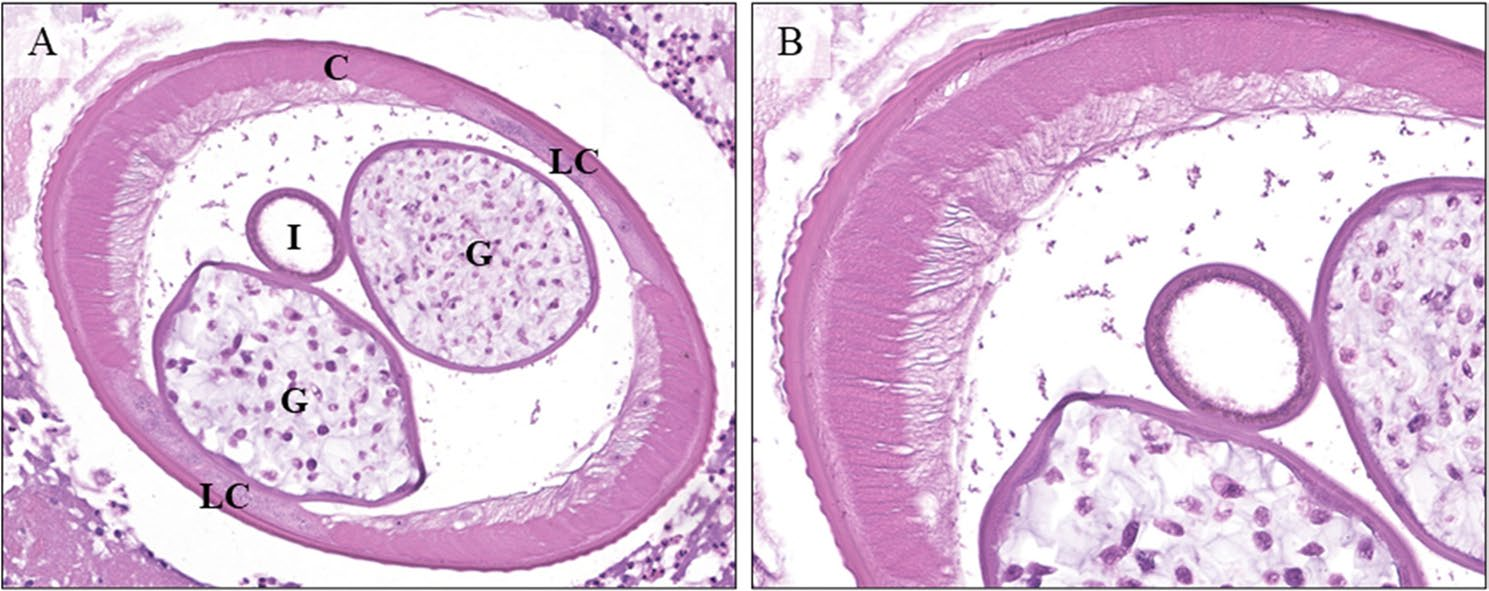
**A** Higher magnification (20X) showing the detail of the multilayered cuticle, the pseudocoelomatic cavity with the intestine and the genital tubules with numerous ovocytes. The main structures of the worm’s body are clearly visible in this image: The cuticle (C) with parallel ridges of muscle fibers, the lateral cords (LC), the intestine (I) and the two genital tubules (G). Several eosinophils surround but do not infiltrate the nematode’s tissues. H&E. B: 40X Detail of the cuticle, one of the most diagnostic features of a nematode, allowing the diagnosis of the genus Dirofilaria. Together with the description of the genital tubules and the size, these morphologic features allow for the diagnosis of *Dirofilaria repens* infection. H&E.

We counted 19 cross sections of the body of the worm, with a maximum transversal diameter of 456 μm (Fig. 5).

The morphological features were mostly intact, a sign that the worm was either alive or recently dead at the time of excision [13].

Following the diagnosis the patient underwent further laboratory testing, including PCR and specific IgG dosing on serum for *Dirofilaria* and for *Trichinella*, which were negative. Other blood parameters and IgE dosing were within the norm for the age of the patient.

The postoperative course was uneventful, with no complications or recurrences observed during follow-up.

## Discussion

*Dirofilaria* infections are endemic in cats and dogs of the Mediterranean region [21], trasmission to humans occurs via arthropod vectors, primarly mosquitos in the genera *Anopheles*, *Aedex and Culex *[6]. However, humans are an accidental host, and the nematodes do not reach sexual maturity [24]. The most frequent presentation of *Dirofilaria repens* infection is that of a subcutaneous nodule of the ocular adnexa, which is often painful and inflamed[2, 5, 12], but other Dirofilaria species can affect different districts, such as the pulmonary vascular tree or the epididymis, or even cause systemic disease [2, 5, 11]. Indeed, emerging data, as previously stated, prove that microfilaremia can occur in humans. Interestingly, data gathered in dogs has shown a possible link between the severity of microfilaremia and overall clinical condition of the infected host [22].

Histological examination and description of the parasite by morphology is usually sufficient for a diagnosis, and clinical suspicion is not generally high enough to warrant specific laboratory workup before surgery, which would include PCR on fresh bioptic samples and serum specific antibody titer, although both could be unsuccessful in the case of older lesions, since these contain only parasitic remains *(*Pampiglione et al., [13]. When preserved the features include a distinctive layered cuticle with thick parallel ridges, in bands perpendicular to the body axis. The two bands of parallel ridges (representing the smooth cell musculature) are separated by lateral cords characterized by an amorphous material and few nuclei [11]. The pseudocoelomatic cavity contains a single small tube representing the esophagus and intestine and larger ones representing the reproductive system: a double one for females and a single one for males [5].

Infections with parasites from the genus *Dirofilaria* are rare, but some regions in the mediterranean basin, such as Italy and southern France, have a relatively higher number of cases [14], Although rare, there is evidence that infection’s incidence is increasing. Potential causes are the climate change which predisposes to the spread of the vector, closer contact with canine and feline pets, and higher awareness by physicians of this disease [12, 19].

Differential diagnosis is therefore crucial to exclude neoplastic or cystic lesions, as in our case, or infection by the more dangerous species of the genus.

Integrated and digitalized imaging in ophthalmic pathology is crucial in the diagnostic process of such challenging and rare cases. This approach could aid in the more accurate diagnosis of zoonoses, as they increasingly pose a challenge for care providers in the age of advancing climate change and increasing mobility of populations.

Furthermore, as radiologic clues are subtle and aspecific in these diseases, close collaboration and consultation of expert opinions is all the more relevant [9]

Clinicians and pathologists operating in this field increasingly approach the workflow in an integrated way in many key areas, such as cancer margin status evaluation [7] and initial assessment of peri-ocular lesions [1].

A more widespread use of digital imaging in ocular pathology not only would allow for these beneficial collaborations between radiologists, surgeons, and pathologists, but would ensure more expedite and accurate diagnoses in rare disease, such as the one presented here, facilitating consultation of experts [15].

Integrated approaches have been proven useful not on only in ophthalmology, but in many other contexts of care, such as the grading of prostate cancer, thus allowing more accurate and less impactful prognostication [8], They also allow for the implementation of powerful computational pathology tools, aiding, for example, in the evaluation of lymph node involvement in gastric cancer [23].

Additionally, in the specific case of this emerging zoonosis, when diagnostic suspicion is low, the chance to obtain adequate material for molecular evaluation is often lost when the parasite is not isolated live, as in our case, and submitted entirely for evaluation, since, as already stated, fresh bioptic samples are generally required for DNA extraction [13].

The aim of this report was to describe a typical case of a rare ophthalmic lesion, accompanied with radiological imaging, clinical data and digitalized slides, with a summary of the diagnostic features necessary to identify this parasite, and to distinguish it from its potentially more dangerous close relatives, in the context of the rising incidence and spread of the vector for this zoonosis, which is increasingly considered an emerging condition [3, 17].

## Data Availability

Not applicable.
